# On the dynamical aspects of local translation at the activated synapse

**DOI:** 10.1186/s12859-020-03597-0

**Published:** 2020-09-14

**Authors:** Tamara M. Khlebodarova, Vladislav V. Kogai, Vitaly A. Likhoshvai

**Affiliations:** 1 Department of Systems Biology, Institute of Cytology and Genetics, Siberian Branch of the Russian Academy of Sciences, Novosibirsk, 630090 Russia; 2 Novosibirsk State University, Novosibirsk, 630090 Russia

**Keywords:** FMRP, Local translation, Synapse, mTOR pathway, Modeling, Complex dynamics

## Abstract

**Background:**

The key role in the dynamic regulation of synaptic protein turnover belongs to the Fragile X Mental Retardation Protein, which regulates the efficiency of dendritic mRNA translation in response to stimulation of metabotropic glutamate receptors at excitatory synapses of the hippocampal pyramidal cells. Its activity is regulated via positive and negative regulatory loops that function in different time ranges, which is an absolute factor for the formation of chaotic regimes that lead to disrupted proteome stability. The indicated condition may cause a number of neuropsychiatric diseases, including autism and epilepsy. The present study is devoted to a theoretical analysis of the local translation system dynamic properties and identification of parameters affecting the chaotic potential of the system.

**Results:**

A mathematical model that describes the maintenance of a specific pool of active receptors on the postsynaptic membrane via two mechanisms – de novo synthesis of receptor proteins and restoration of protein function during the recycling process – has been developed. Analysis of the model revealed that an increase in the values of the parameters describing the impact of protein recycling on the maintenance of a pool of active receptors in the membrane, duration of the signal transduction via the mammalian target of rapamycin pathway, influence of receptors on the translation activation, as well as reduction of the rate of synthesis and integration of de novo synthesized proteins into the postsynaptic membrane – contribute to the reduced complexity of the local translation system dynamic state. Formation of these patterns significantly depends on the complexity and non-linearity of the mechanisms of exposure of de novo synthesized receptors to the postsynaptic membrane, the correct evaluation of which is currently problematic.

**Conclusions:**

The model predicts that an increase of “receptor recycling” and reduction of the rate of synthesis and integration of de novo synthesized proteins into the postsynaptic membrane contribute to the reduced complexity of the local translation system dynamic state. Herewith, stable stationary states occur much less frequently than cyclic states. It is possible that cyclical nature of functioning of the local translation system is its “normal” dynamic state.

## Introduction

Existing views on the importance of a stable proteome for the formation of synaptic plasticity (see reviews: [1–3]) and associated learning and memory processes bring impairments in the local translation at the activated synapse to the fore as a cause of several neuropsychiatric diseases (e.g., autism, epilepsy). It is known that one of the manifestations of epilepsy [4, 5] and autistic pathologies [6–8] is increased activity of the mammalian target of rapamycin (mTOR) signaling pathway, which is the central link in the regulation of local cap-dependent translation at the synapse. It has been theoretically shown that dynamic relationship between activation and suppression of local translation at glumatergic synapses in response to their activation may result in formation of complex dynamics of postsynaptic protein synthesis in the area of physiological functioning of this system, and, therefore, may impair proteome stability at the activated synapse [9].

As it turned out, peculiarities of the regulation of local translation associated with the activation of the mTOR signaling pathway determine the high chaotic potential of the system [9, 10]. It was found that it depends on the ratio between activation and block of the activity of the RNA-binding protein FMRP (Fragile X Mental Retardation Protein) [9, 10], a key regulator of dendritic mRNA translational efficiency [11–13]. That is, factors destabilizing the local translation system at the activated synapse are inherent in its regulatory mechanisms, so it is not surprising that disrupted mTOR activity, which can occur both due to mutations and various external influences, is one of the manifestations of autistic disorders [6–8] and epilepsy [4, 5].

**Fig. 1:**
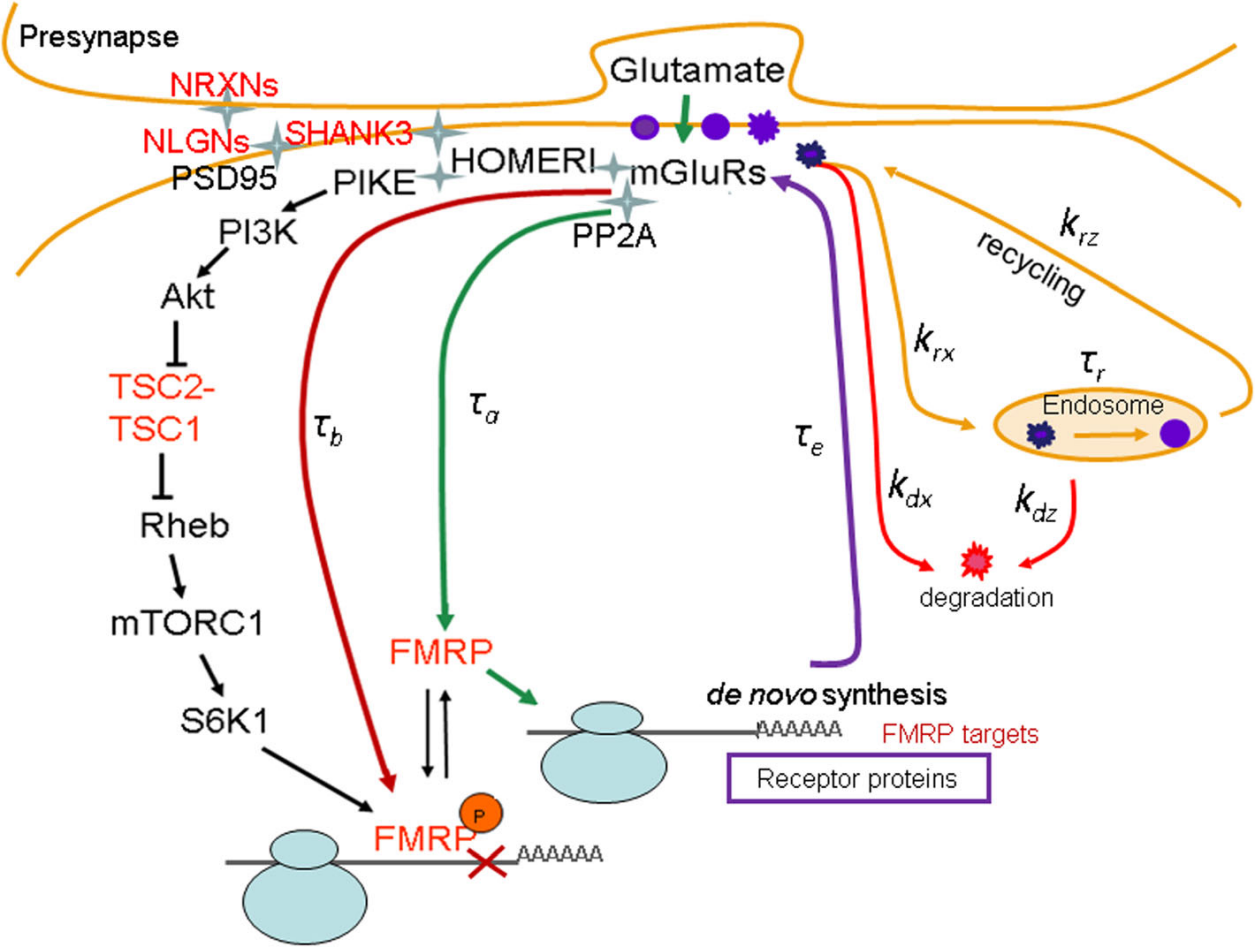
Scheme of processes that maintain a certain receptor density on the postsynaptic membrane at the activated synapse. The right hand side shows signaling pathways for the regulation of FMRP-dependent translation through PP2A phosphatase and S6 kinase. On the left is the recycling of receptors through endocytosis (marked in light brown). The delay parameters *τ*_*а*_ and *τ*_*b*_ describe the time of activation (green arrow) and inhibition (dark red arrow) of de novo protein synthesis, τ_e_ is the time of protein translation and inclusion into the receptor complex (violet arrow), and τ_r_ is the time the receptor protein is localized in the endosome. The *k*_*rz*_ constant describes the efficiency of receptor recycling, the *k*_*rx*_ and *k*_*dx*_ constants are the rates of receptor inclusion into the endosome and its degradation, respectively; *k*_*dz*_ constant is the rate of receptor proteins degradation in the endosome. mGluR (metabotropic glutamate receptor); FMRP (fragile X mental retardation protein) – dendritic mRNA-binding protein; NRXNs (neurexins) – presynaptic membrane proteins; NLGNs (neuroligins), SHANK3 (SH3 domain and ankyrin repeat-containing protein), PCD-95 (postsynaptic density protein 95), HOMER – postsynaptic membrane proteins; PIKE (phosphoinositide-3 kinase enhancer), PI3K (phosphatidylinositol 3-kinase), Akt (protein kinase B), TSC1 (tumor suppressor hamartin), TSC2 (tumor suppressor tuberin), Rheb (Ras homolog enriched in brain), S6 kinase – mTOR (mammalian target of rapamycin) signaling pathway. Red color marks the names of proteins whose gene mutations are associated with neurodegenerative diseases

Moreover, it was shown that practically no areas of stable stationary states in the dynamics of postsynaptic density (PSD) proteins synthesis in the physiological area of functioning of the local translation system [9, 14]. Due to the lack of quantitative data on the structural and functional organization of the synapse, it is currently not possible to define the parametric space of stable functioning of the local translation system. At the same time, it is possible that previously obtained results illustrate the simplicity of the model, which did not consider cellular processes that were developed in the course of evolution for the synaptic stabilization. It should be noted that during previous analysis of the intrinsic properties of the dendritic mRNA translation regulation, we did not consider the maintenance of the postsynaptic membrane receptor density associated with protein recycling. We assumed that maintenance of the receptor density at the activated synapse occurred only due to de novo synthesized proteins, which is a clear simplification of the model [9, 10, 14]. Recycling of receptor proteins at the synapse is implemented through the mechanisms of their endo- and exocytosis and is closely related to the ubiquitin-dependent protein degradation during formation of synaptic plasticity [15, 16]. That suggests the influence of the recycling pool of receptor proteins at the activated synapse on the dynamics of their de novo synthesis. Based on this, we developed a model, in which the maintenance of a certain density of receptor proteins at the postsynaptic membrane is achieved both due to de novo protein synthesis as well as a result of protein cyclization; and investigated the impact of the receptor recycling pathway on the dynamic state of the local translation system at the activated synapse. The regulatory circuit described in the model is presented in Fig. 1.

Analysis of the model revealed that, indeed, increased contribution of recycling to the maintenance of receptor protein density on the membrane results in the domination of simple modes, oscillatory or stationary, in the dynamics of de novo receptor protein synthesis. However, the type of the mode is largely determined by the complexity and nonlinearity of the mechanism of exposure of de novo synthesized proteins to the postsynaptic membrane, the correct evaluation of which is currently challenging. Therefore, the interpretation of the research results is limited, but it creates certain prerequisites for the assumption that cyclical nature of the local translation system at the activated synapse under certain conditions may be its “normal” dynamic state.

## Results

Due to the non-linearity and relatively high complexity of the model describing the system for maintaining a certain pool of active receptors on the postsynaptic membrane of the activated synapse, which includes at least two processes - de novo receptor protein synthesis and restoration of receptor sensitivity during recycling (see Fig. 1), a comprehensive analysis of the model over the space of physiological parameter values is not possible. Therefore, to assess the dynamic properties of the model, we used the method of in silico computational experiments with various specific physiological sets of parameter values. Obtained numerical data were used to develop biological interpretations.

### Influence of the recycling parameter *k*_*rz*_ on the system (1) dynamics at the minimum values of the delay parameters

**Fig. 2:**
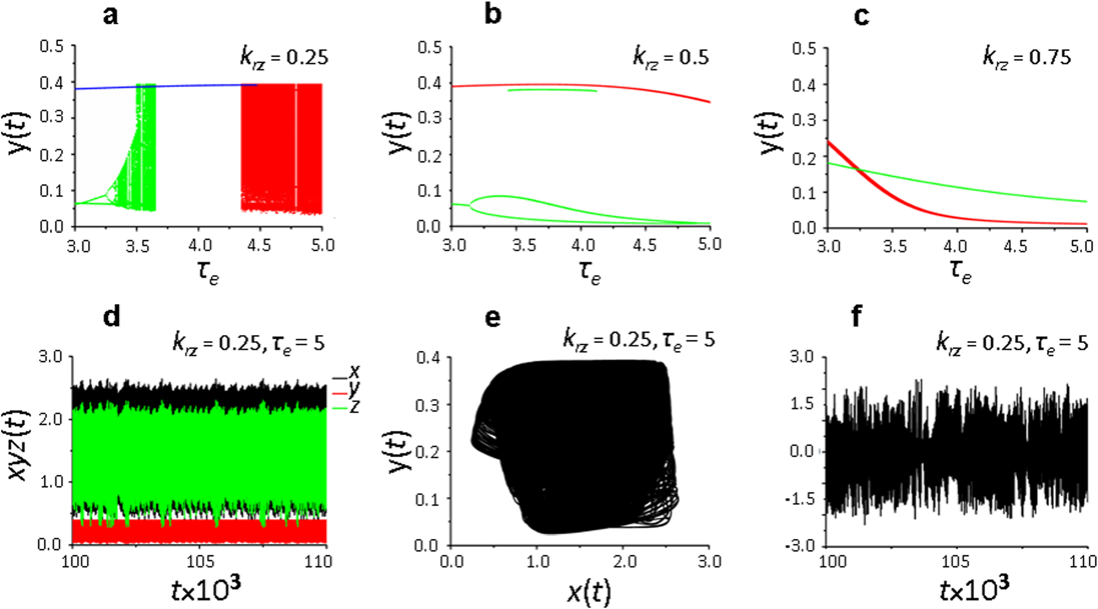
System (1) solutions depending on the *k*_*rz*_ parameter, which determines the contribution of recycling to the size of the total pool of receptor proteins. (**a**–**c**) – bifurcation diagram constructed at the intersection of the trajectory (*х*(*t*), *y*(*t*)) with the Poincaré map *х*(*t*) = 2 in the phase space (*x,y,z*), *τ*_*е*_ = 3–5: *k*_*rz*_ = 0.25 (**a**), *k*_*rz*_ = 0.5 (**b**), *k*_*rz*_ = 0.75 (**c**); (**d**) – dependence of *x,y,z* on *t*, *k*_*rz*_ = 0.25, *τ*_*е*_ = 5; (**e**) – type of solution in space (*x,y*), *k*_*rz*_ = 0.25, τ_е_ = 5; (**f**) – difference *x*_1_(*t*) − *x*_2_(*t*) between the solutions of the two Cauchy problems with the initial data differing by 0.000001, *k*_*rz*_ = 0.25, *τ*_*е*_ = 5. Values of the delay parameters *τ*_*а*_ = 1, *τ*_*b*_ = 2, *τ*_*r*_ = 3, values of the remaining parameters are shown in the basic set (2). Different colors indicate different branches of the diagram

**Fig. 3:**
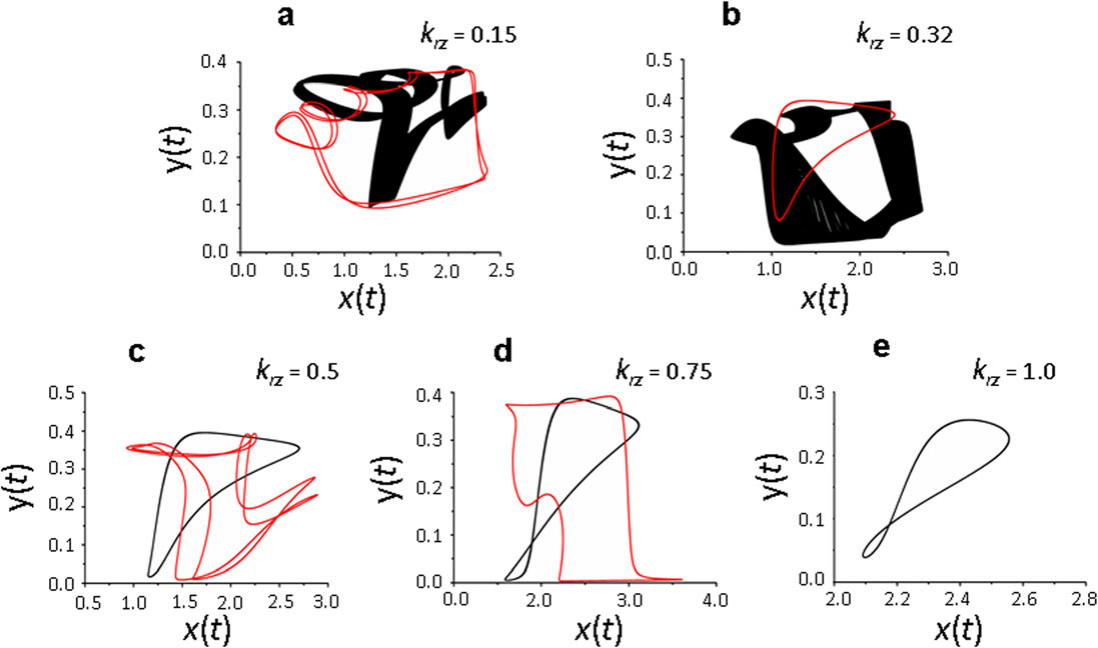
Phase portrait of the solutions of system (1) in the space (*x,y*) depending on the parameter *k*_*rz*_. Periodic solution and chaos at *k*_*rz*_ = 0.15 (**a**) and *k*_*rz*_ = 0.32 (**b**), periodic solutions at *k*_*rz*_ = 0.5 (**с**) and *k*_*rz*_ = 0.75 (**d**), periodic solution at *k*_*rz*_ = 1 (**e**). Values of the delay parameters *τ*_*а*_ = 1, *τ*_*b*_ = 2, *τ*_*r*_ = 3, *τ*_*e*_ = 5, values of the remaining parameters are shown in the basic set (2). Different colors indicate different branches of the diagram

We have previously shown that with a decrease in the ratio of delay parameters characterizing the time of translation activation (*τ*_*а*_) and supression (*τ*_*b*_), the chaotic potential of the system increases [10]. Therefore, analysis of the impact of the receptor protein recycling on their de novo synthesis at the activated synapse was carried out with minimal, physiologically justified values of *τ*_*а*_ and *τ*_*b*_ delay parameters, as well as *τ*_*r*_ and *τ*_*е*_ parameters, which determine the time of receptor recycling, de novo synthesis of receptors and their incorporation into the membrane. Solutions of model (1) depending on the contribution of recycling to the size of the total pool of receptor proteins (parameter *k*_*rz*_) are presented in Figs. 2 and 3.

It follows from Fig. 2a that if 25% of the total pool of recycled receptors is returned from the endosome to the membrane, the multiplicity of attractors (shown in different colors) is a characteristic feature of the dynamic state of the local translation system at the synapse. The complexity of the attractors significantly depends on the value of the parameter *τ*_*e*_. Three different attractors have been observed: a simple cycle for the *τ*_*e*_ value lying in the interval from 3 to ~ 4.47 (a line on the Poincare map) and two attractors with complex vibrational-irregular dynamics. The attractor observed for the *τ*_*e*_ value lying in the interval from 3 to ~ 3.65 is primarily cyclic and the doubling of cycles occurs with increasing *τ*_*e*_, which ultimately leads to the formation of chaotic dynamics according to the Feigenbaum scenario [17, 18]. Following aspects suggest the chaotic state of the vibrational-irregular attractor in the *τ*_*е*_ interval from ~ 4.35 to 5: bifurcation diagram (Fig. 2a), dependence of the parameters *x, y, z* on *t* (Fig. 2d), phase portrait of the solution (Fig. 2e), and the sensitivity of the solution of the Cauchy problem to initial data differed by 0.000001 (Fig. 2f). As follows from Fig. 2b, c, with an increase in the contribution of recycled receptors to the total pool of active receptors up to 50–75%, the chaotic dynamics of de novo protein synthesis is transformed into cyclic, but the multiplicity of attractors remains.

The same conclusion follows from the analysis of the nature of changes in the phase portrait of the solutions of the system of equations (1) depending on *k*_*rz*_ (Fig. 3a – e). Multiplicity of solutions and presence of a chaotic solution are clearly visible at *k*_*rz*_ = 0.15 and *k*_*rz*_ = 0.32 (Fig. 3a, b); the solution becomes periodic at *k*_*rz*_ = 0.5– 0.75 (Fig. 3c, d). The system of equations (1) has only one cyclical solution at *k*_*rz*_ = 1, that is, when the entire pool of recycled proteins (in our case - 90% of the active receptors exposed to the membrane (*k*_*rx*_ = 0.9)), returns to the membrane.

Henсe, a clear dependence of the dynamic state of the local translation system on the contribution of recycling to the total pool of active receptors is observed for the investigated region of delay parameters (*τ*_*е*_ = 3–5, *τ*_*a*_ = 1,  *τ*_*b*_ = 2,  *τ*_*r*_ = 3): with an increase in the recycling parameter *k*_*rz*_, a transition from chaotic to cyclic regime is observed, that is, the dynamic state of the system becomes more simple.

### Influence of delay parameters *τ*_*r*_ and *τ*_*e*_ on the dynamics of the system (1)

Parameter *τ*_*e*_ characterizes the translation elongation time and the time of de novo synthesized protein exposure to the membrane. We have previously shown that, within the time intervals, during which regulation of local translation at real synapse occurs via positive and negative feedback loops [19, 20], an increase in the *τ*_*e*_ value affects the dynamics of the system in a similar way as shown above for the recycling parameter *k*_*rz*_, that is, with an increase in *τ*_*e*_ value (*τ*_*e*_ *>* 5), a transition from a chaotic regime to a cyclic one is observed [9].

To analyze the influence of the combined effect of protein recycling parameters (*k*_*rz*_ and *τ*_*r*_) and *τ*_*e*_ parameter, a series of studies of model (1) solutions was carried out at higher, physiologically justified values of the delay parameters *τ*_*r*_ = 10–15 and *τ*_*e*_ = 15–20.

**Fig. 4:**
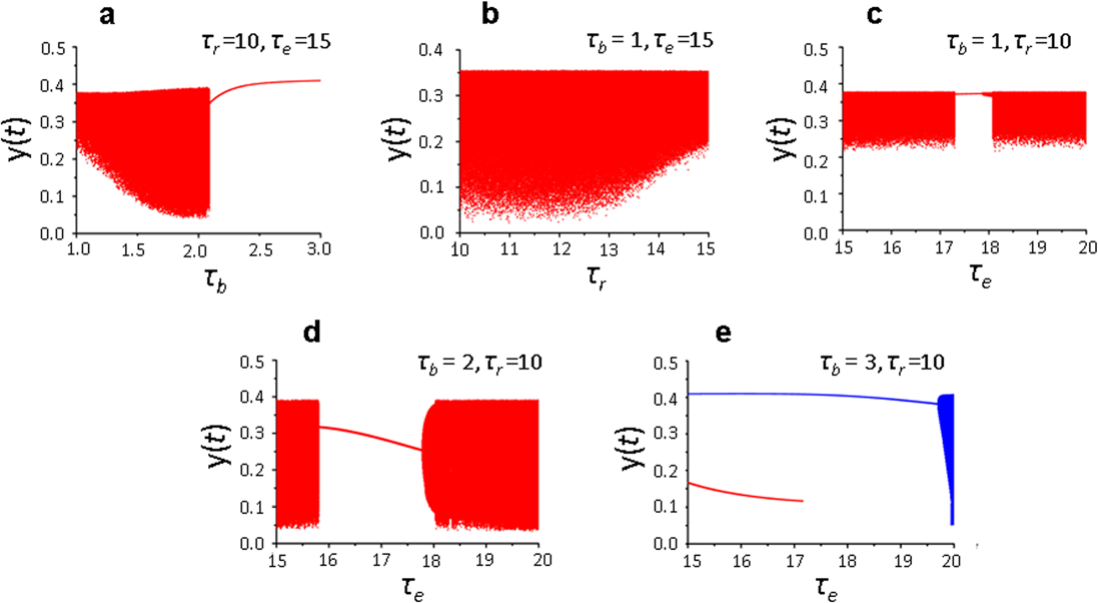
System (1) solutions depending on the delay parameters τ. Bifurcation diagram constructed at the intersection of the trajectory (*х*(*t*), *y*(*t*)) with the Poincaré map *х*(*t*) = 1 in the phase space (*x,y,z*) at *τ*_*a*_ = 1, *k*_*rz*_ = 0.2. Parameter values: (**a**) *τ*_*e*_ = 15, *τ*_*r*_ = 10; (**b**) *τ*_*b*_ = 1, *τ*_*e*_ = 15; (**с**) *τ*_*b*_ = 1, *τ*_*r*_ = 10; (**d**) *τ*_*b*_ = 2, *τ*_*r*_ = 10; (**e**) *τ*_*b*_ = 3, *τ*_*r*_ = 10*.* Values of the remaining parameters are shown in the basic set (2). Different colors indicate different branches of the diagram

**Fig. 5:**
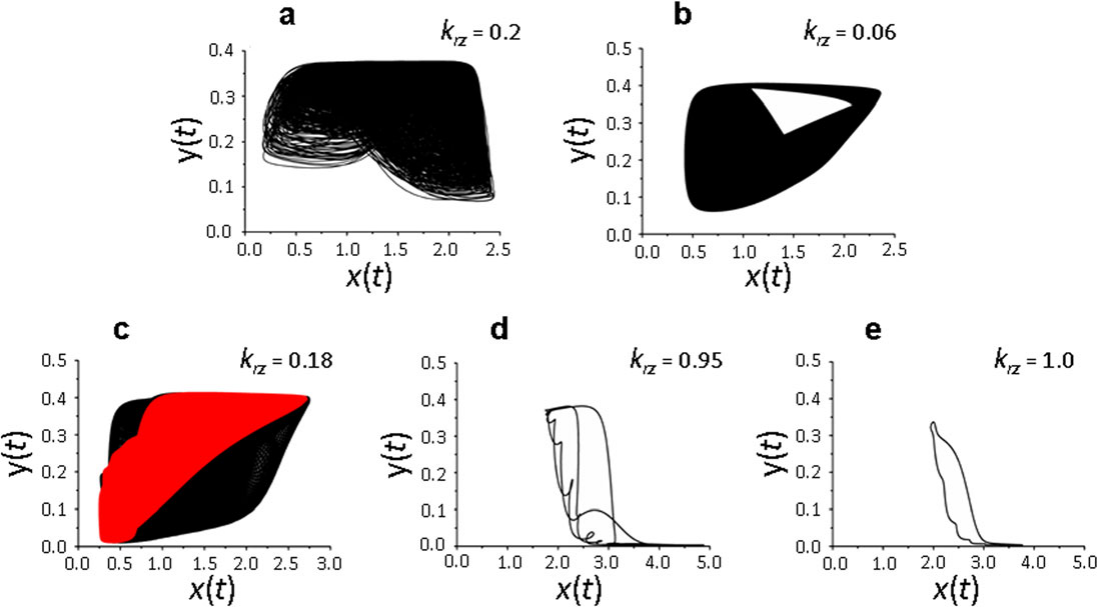
Phase portrait of the system (1) solutions in the space (*x,y*) depending on the parameter *k*_*rz*_. (**a**) chaotic solution at *τ*_*b*_ = 1_,_ τ_e_ = 15, *k*_*rz*_ = 0.2, (**b**, **c**) quasi-periodic solutions at *k*_*rz*_ = 0.06 (**b**) and *k*_*rz*_ = 0.18 (**c**, **d**, **e**) periodic solutions at *k*_*rz*_ = 0.95 (**d**) and *k*_*rz*_ = 1 (**e**). Values of the delay parameters *τ*_*a*_ = 1, *τ*_*r*_ = 10 (**a**-**f**)_,_ *τ*_*b*_ = 3_,_ *τ*_*e*_ = 20 (**b**-**e**), values of the remaining parameters are shown in the basic set (2). Different colors indicate different branches of the diagram

The research results are shown in Figs. 4 and 5. It can be seen that complex dynamics of protein synthesis is observed in the range of delay parameters *τ*_*r*_ = 10–15 and *τ*_*e*_ = 15–20 (Fig. 4b-d), which is represented by both chaotic and quasi-periodic solutions (Fig. 5a–c). This complex dynamics becomes periodic with increasing value of the parameter *τ*_*b*_ (Fig. 4a, e), which is consistent with previous results [9, 10], as well as at high values of the parameter *k*_*rz*_. Such regularity is demonstrated above with the minimum values of the delay parameters (Fig. 5d, e).

Thus, within the framework of the model (1), which considers the contribution of receptor recycling to maintaining receptor density on the membrane, we did not observe qualitative changes in the dynamics of the local translation system with the delay parameters *τ*_*r*_ = 10–15 and *τ*_*e*_ = 15–20 compared to the dynamic modes described above with *τ*_*r*_ = 3 and *τ*_*e*_ = 3–5 (compare Figs. 2a and 4d).

The question arises, do other varying parameters of the local translation system affect the manifestation of the revealed pattern?

**Fig. 6:**
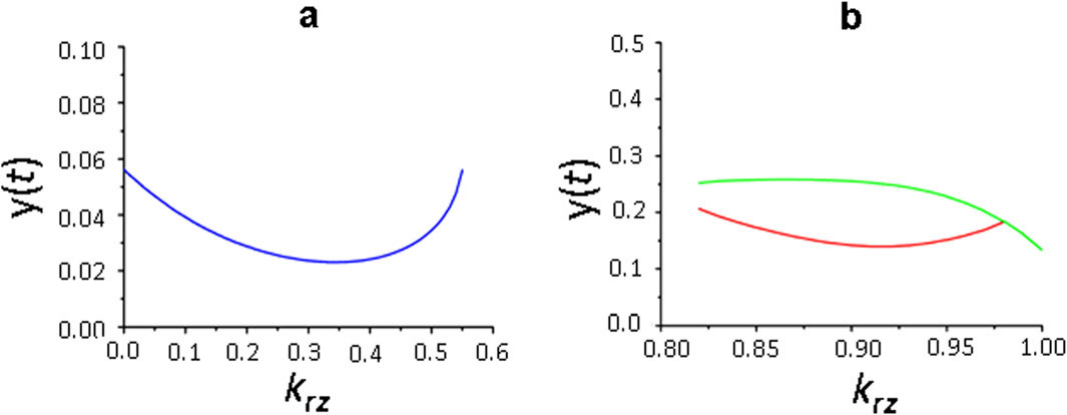
Solutions of system (1) at minimum (**a**) and maximum (**b**) values of varying parameters depending on the parameter *k*_*rz*_. Bifurcation diagram constructed at the intersection of the trajectory (*х*(*t*), *y*(*t*)) with the Poincaré map *х*(*t*) = 2 in the phase space (*x,y,z*): (**а**) – periodic solutions at *k*_*rz*_ < 0.55, stationary solutions at *k*_*rz*_ > 0.56, parameter values: *h*_*x*_ = 1, *h*_*b*_ = 5, *k*_*x*_ = 10, *k*_*b*_ = 100, *K*_*a*_ = 1; (**b**) – stationary solutions at *k*_*rz*_ < 0.8, periodic solutions at *k*_*rz*_ > 0.82, parameter values: *h*_*x*_ = 3, *h*_*b*_ = 15, *k*_*x*_ = 40, *k*_*b*_ = 200, *K*_*a*_ = 3. Values of the delay parameters *τ*_*а*_ = 1, *τ*_*b*_ = 2, *τ*_*r*_ = 3, *τ*_*e*_ = 3, values of the remaining parameters are shown in the basic set (2)

We examined five parameters: *h*_*b*_ and *h*_*x*_ describe the complexity and nonlinearity of the FMRP phosphorylation and exposure of the synthesized proteins to the membrane; *k*_*b*_ and *k*_*x*_ constants determine the maximum rate of signal-dependent FMRP phosphorylation and the rate of receptor synthesis and incorporation into the membrane; *K*_*a*_ parameter by definition determines the effect of glutamatergic receptors on activation of local translation. We found that both with the minimum values of these varying parameters and those close to the maximum values (according to the above estimates), only stationary and periodic solutions are observed for system (1), but the area of their manifestation depends on the recycling parameter *k*_*rz*_ (Fig. 6). In the first case, at low values of the *k*_*rz*_ parameter, periodic solutions were observed and became stable stationary solutions at *k*_*rz*_ > 5.6 (Fig. 6a), that is, the dynamic state of the local translation system was simplified. In the second case, on the contrary, with increasing *k*_*rz*_, a transition from more simple stationary solutions to more complex periodic ones, occurred (Fig. 6b). However, calculations showed that in this case stationary solutions for *k*_*rz*_ = 0 and *k*_*rz*_ = 0.5 were zero, that is, most likely, system (1) functioned only in an oscillatory mode and only with a significant contribution of receptor recycling (*k*_*rz*_ > 0.82) to the formation of a general pool of receptor proteins (Fig. 6b).

Which of the studied parameters so radically affect the dynamic state of the local translation system at the activated synapse at high and low values of *k*_*rz*_ recycling parameter?

### Behavior of the model (1) depending on the influence of glutamatergic receptors on the activation of local translation (*K*_*a*_)

It should be noted that basic set of parameters (2) was found to be close to the set of parameter values, which we designated above as “maximum”, with the exception of the *K*_*a*_ parameter. Therefore, we began the study of the dynamic state of the system (1) with the analysis of the *K*_*a*_ parameter, which determines the effectiveness of the influence of glutamatergic receptors on the activation of local translation.

**Fig. 7:**
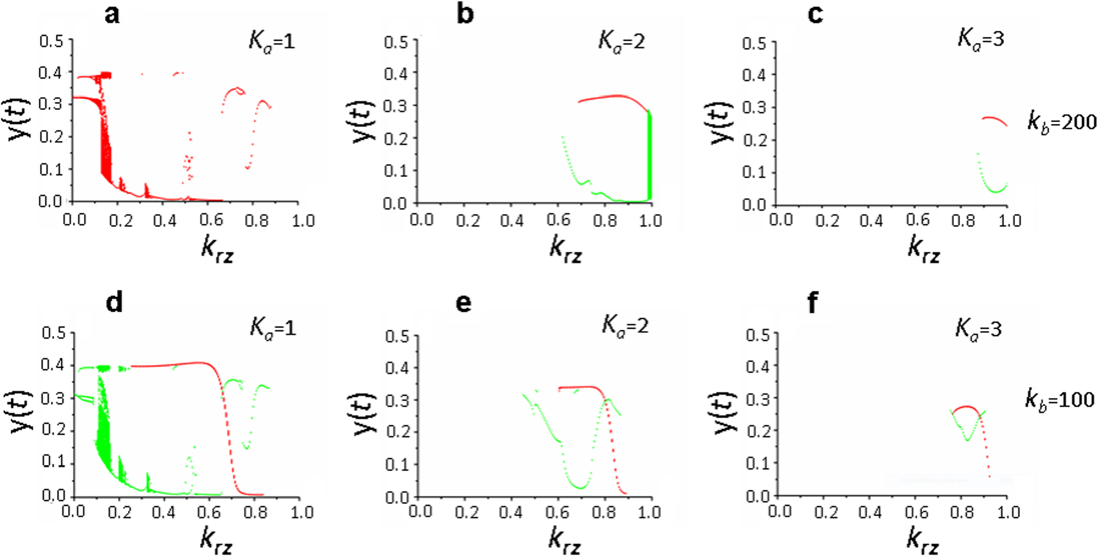
Solutions of the system of equations (1) with the “maximum” values of the varying parameters depending on the parameters *k*_*rz*_ and *K*_*a*_. Bifurcation diagram constructed at the intersection of the trajectory (*х*(*t*), *y*(*t*)) with the Poincaré map *х*(*t*) = 2 in the phase space (*x,y,z*): (**a**,**d**) *K*_*a*_ = 1, (**b**,**e**) *K*_*a*_ = 2., (**c**,**f**) *K*_*a*_ = 3. Parameter values *τ*_*а*_ = 1, *τ*_*b*_ = 2, *τ*_*r*_ = 3, *τ*_*e*_ = 3, *k*_*b*_ = 200 (**a**-**c**), *k*_*b*_ = 100 (**d**-**f**), values of the remaining parameters are shown in the basic set (2). Two cyclic solutions at *k*_*rz*_ = 1, *K*_*a*_ = 3, *k*_*b*_ = 100 (**f**) do not match the selected Poincaré plane *x*(*t*) = 2

It was found that with “maximum” values of the varying parameters *h*_*x*_ = 3, *h*_*b*_ = 20, *k*_*x*_ = 40 and *k*_*b*_ = 200 (Fig. 7a-c), *K*_*a*_ parameter significantly affects the complexity of the dynamic regime of the system (1).

However, it can be seen that, depending on the *k*_*rz*_ parameter value, this influence is multidirectional: when *k*_*rz*_ < 0.8, a transition from complex dynamic regimes to simple stationary solutions occurs with increasing *K*_*a*_ parameter value, while when *k*_*rz*_ values are high, on the contrary, there is a transition from stationary solutions to more complex, periodic ones. It was also found that two times reduction in the maximum FMRP phosphorylation rate, *k*_*b*_ = 100 (Fig. 7d-f), does not lead to qualitative changes in the picture observed below and its dynamics depending on the *K*_*a*_ parameter (compare pairs of Fig. 7a and d, b and e, c and f). Calculations showed that with *K*_*a*_ = 3 and *k*_*rz*_ = 1 in system (1), regardless of the *k*_*b*_ parameter value, two solutions are observed and they are both cyclic, with *k*_*rz*_ = 0 and *k*_*rz*_ = 0.5 the solutions are stationary, but they are zero.

The conclusion on the relatively weak influence of the *k*_*b*_ parameter on the dynamic behavior of the system (1) is retained for other values of varying parameters (see Additional files 1 and 2, Fig. S1 and S2). Further analysis have shown that the revealed pattern related to the influence of the *K*_*a*_ parameter on the dynamic state of the system (1) substantially depends on the *h*_*x*_ parameter value, which determines the non-linearity and complexity of the mechanism of exposure of de novo synthesized proteins to the postsynaptic membrane, the correct evaluation of which is currently difficult. Therefore, we conducted a detailed analysis of the effect of *h*_*x*_ parameter on the behavior of the system (1).

### Influence of the *h*_*x*_ parameter on the system (1) dynamics

**Fig. 8:**
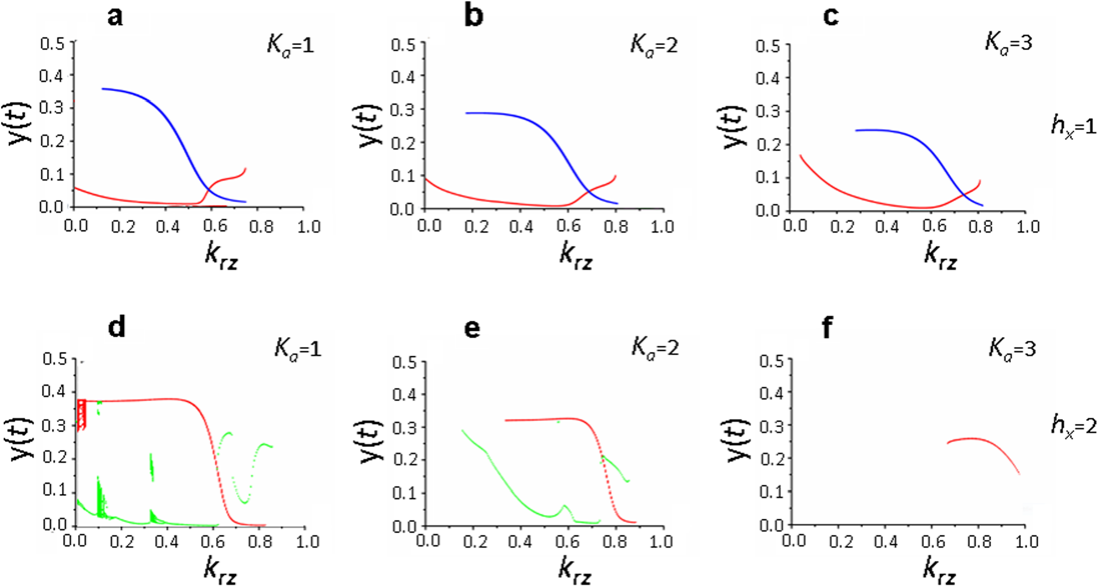
System (1) solutions depending on the parameters *k*_*rz*_ and *K*_*a*_ and at different values of the *h*_*x*_ parameter, which determines the complexity of the mechanism of exposure of de novo synthesized proteins to the postsynaptic membrane. Bifurcation diagram constructed at the intersection of the trajectory (*х*(*t*), *y*(*t*)) with the Poincaré map *х*(*t*) = 2 in the phase space (*x,y,z*). Parameter values: *h*_*x*_ = 1, *h*_*b*_ = 10, *k*_*x*_ = 10 (**a**-**c**); *h*_*x*_ *=* 2, *h*_*b*_ = 15, *k*_*x*_ = 20 (**d**-**f**); *τ*_*а*_ = 1, *τ*_*b*_ = 2, *τ*_*r*_ = 3, *τ*_*e*_ = 3, *k*_*b*_ = 100. Values of the remaining parameters are shown in the basic set (2). Different colors indicate different branches of solutions. (**d**, **e**) the solution is periodic at *k*_*rz*_ = 1, however it does not intersect with the Poincaré plane *х*(*t*) = 2

Analysis of the influence of the *h*_*x*_ parameter on the system (1) behavior presented below and in Additional file 1 have shown that with *h*_*x*_ = 1, *τ*_*b*_/*τ*_*a*_ = 2 and different values of the parameters *h*_*b*_, *k*_*x*_, and *K*_*a*_, main solutions of the system (1) are periodic solutions which become stationary with an increase in the recycling parameter; and *K*_*a*_ parameter has practically no effect on this dependence (Fig. 8a-c). Specific periodic solutions of equation (1) are determined by the parameters *h*_*b*_ and *k*_*x*_, however, no explicit pattern associated with the change of these parameters was found (see Additional file 3, Fig. S3).

When *h*_*x*_ *=* 2, various dynamic states are formed in system (1), including chaotic ones, which, with an increase in the *K*_*a*_ value, become simple, cyclic and stationary states (Fig. 8d-f, Table 1, see also Additional files 1 and 4, Fig. S1 and S4). This pattern is quite pronounced throughout the entire range of variation of the *h*_*b*_ parameter and at *k*_*x*_ = 10 and *k*_*x*_ = 20.

**Table 1:** Model (1) solutions at *h*_*x*_ = 2, which determines the non-linearity and complexity of the process of exposure of de novo synthesized proteins to the postsynaptic membrane, depending on the values of the varying parameters *K*_*a*_, *h*_*b*_, *k*_*x*_ and with varying contribution of recycling (*k*_*rz*_) to maintaining a pool of active receptors on the membrane

***h***_***b***_, ***k***_***x***_	***K***_***a***_ = 1			***K***_***a***_ = 2			***K***_***a***_ = 3		
	*k*_*rz*_ = 0	*k*_*rz*_ = 0.5	*k*_*rz*_ = 1	*k*_*rz*_ = 0	*k*_*rz*_ = 0.5	*k*_*rz*_ = 1	*k*_*rz*_ = 0	*k*_*rz*_ = 0.5	*k*_*rz*_ = 1
5, 10	S	S	S	0	0	S	0	0	S
10, 10	S	P	S	0	S	S	0	0	0
15, 10	S	P	P	0	S	P	0	0	S
20, 10	S	P	2P	0	S	2P	0	0	P
5, 20	QP	P	S	0	S	S	0	S	S
10, 20	2P	2P	S	S	2P	S	0	0	S
15, 20	P + C	2P	P	S	2P	P	0	P	P
5, 40	P	P	S	P	P	S	0	P	S
10, 40	P	2P	S	P	2P	S	QP	2P	S
15, 40	P	2P	P	2P	2P	P	C	2P	P

*S* stationary solution, *0* zero solution, *P* periodic solution, *C* chaos, *QP* quasi-periodic solution

**Fig. 9:**
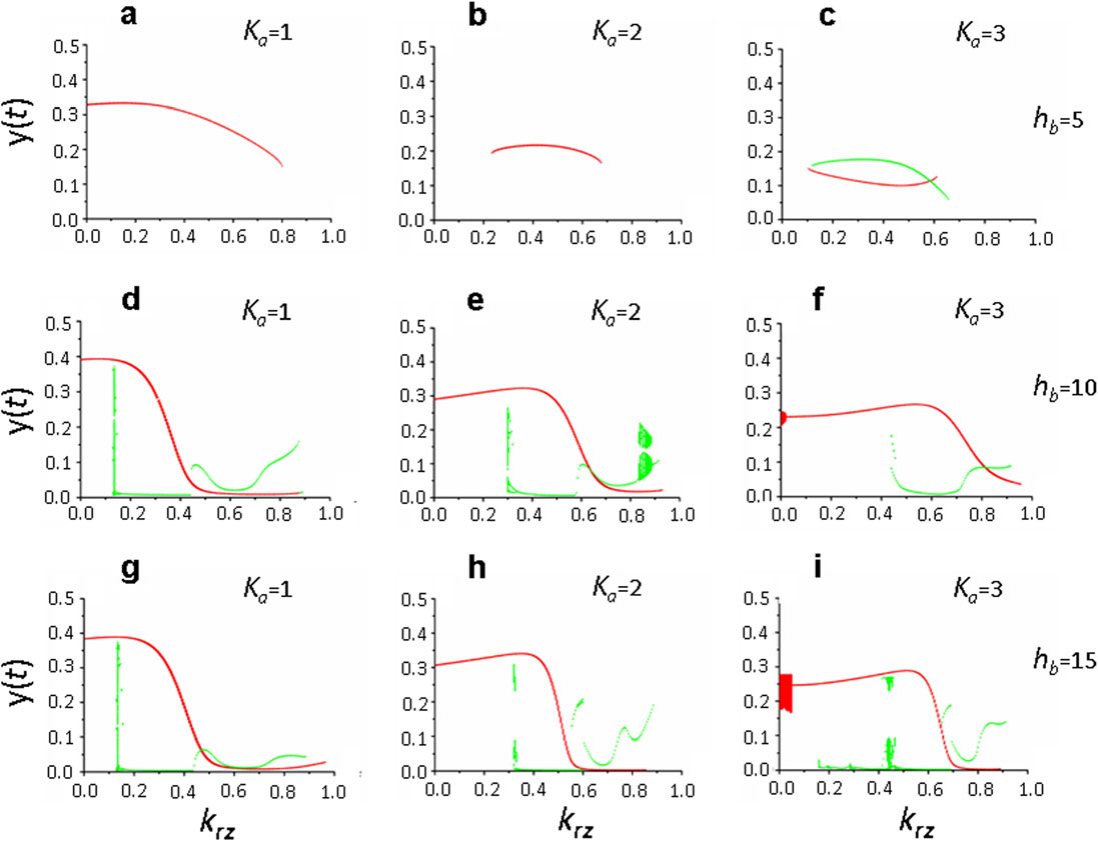
Dynamic regimes of system (1) at maximum rate of FMRP-dependent synthesis of receptor proteins and their incorporation into the membrane (*k*_*x*_ = 40) depending on the efficiency of translation initiation (*K*_*a*_), complexity of the mTOR pathway (*h*_*b*_) and contribution of recycling to the formation of the pool of active receptors (*k*_*rz*_). Bifurcation diagram constructed at the intersection of the trajectory (*х*(*t*), *y*(*t*)) with the Poincaré map *х*(*t*) = 2 in the phase space (*x,y,z*). Parameter values: *h*_*x*_ = 2, *k*_*x*_ = 40, *k*_*b*_ = 100 (**a**-**f**), *k*_*b*_ = 200 (**g**-**i**), *h*_*b*_ = 5 (**a**-**c**), *h*_*b*_ = 10 (**d**-**f**), *h*_*b*_ = 15 (**g**-**i**), *K*_*a*_ = 1 (**a**, **d**, **g**), *K*_*a*_ = 2 (**b**, **e**, **h**), *K*_*a*_ = 3 (**c**, **f**, **i**); *τ*_*а*_ = 1, *τ*_*b*_ = 2, *τ*_*r*_ = 3, *τ*_*e*_ = 3. Values of the remaining parameters are shown in the basic set (2); (**b**, **c**) periodic solution exists at *k*_*rz*_ = 0, however it does not intersect with the Poincaré plane *х*(*t*) = 2. Different colors indicate different branches of the system (1) solutions

At *k*_*x*_ = 40, influence of the *K*_*a*_ parameter on the dynamic state of the system (1) was not detected for any *h*_*b*_ values (Table 1, Fig. 9). In this case, when *h*_*b*_ > 5, system (1) experiences complex dynamic regimes over almost the entire range of changes in the recycling parameter *k*_*rz*_ and transforms into stationary state only at close to maximum values of the *k*_*rz*_ parameter (Fig. 9d-i). At low *h*_*b*_ values, the dynamic regime of system (1) was represented by simple cycles, which transformed into stationary solutions with increased recycling efficiency of receptor proteins (Table 1, Fig. 9a-c).

As for the solutions of system (1) at *h*_*x*_ = 3, main solutions were stationary practically over the whole variety of values of varying parameters considered in this paper. However, specific calculations have shown that stable stationary states are observed only at *k*_*rz*_ values close to unity, which is physiologically unlikely (see Table 2). When ratios between *h*_*b*_ and *k*_*x*_ values are close to “maximum” in system (1), more complex dynamic states arise that transform into stationary and simple cyclic states with increasing *K*_*a*_ (except for *k*_*x*_ = 40). The nature of this pattern depending on the recycling parameter *k*_*rz*_ is shown in Fig. 7.

**Table 2:** Model (1) solutions at parameter *h*_*x*_ = 3, which determines the non-linearity and complexity of the process of exposure of de novo synthesized proteins to the postsynaptic membrane, depending on the values of the varying parameters *K*_*a*_, *h*_*b*_, *k*_*x*_ and with varying contribution of recycling (*k*_*rz*_) to maintaining a pool of active receptors on the membrane

***h***_***b***_, ***k***_***x***_	***K***_***a***_ = 1			***K***_***a***_ = 2			***K***_***a***_ = 3		
	*k*_*rz*_ = 0	*k*_*rz*_ = 0.5	*k*_*rz*_ = 1	*k*_*rz*_ = 0	*k*_*rz*_ = 0.5	*k*_*rz*_ = 1	*k*_*rz*_ = 0	*k*_*rz*_ = 0.5	*k*_*rz*_ = 1
5, 10	0	0	S	0	0	S	0	0	0
10, 10	0	0	S	0	0	S	0	0	0
15, 10	0	0	S	0	0	S	0	0	S
20, 10	0	0	P	0	0	S	0	0	S
15, 20	0	S	P	0	0	S	0	0	S
20, 20	0	S	2P	0	0	2P	0	0	S
15, 40	P	2P	S	0	P	S	0	0	P
20, 40	2P	2P	S	0	P	S	0	0	P

*S* stationary solution, *0* zero solution, *P* periodic solution, *2P* two periodic solutions

### Influence of the parameters *h*_*b*_ and *k*_*x*_ on the system (1) dynamics

**Fig. 10:**
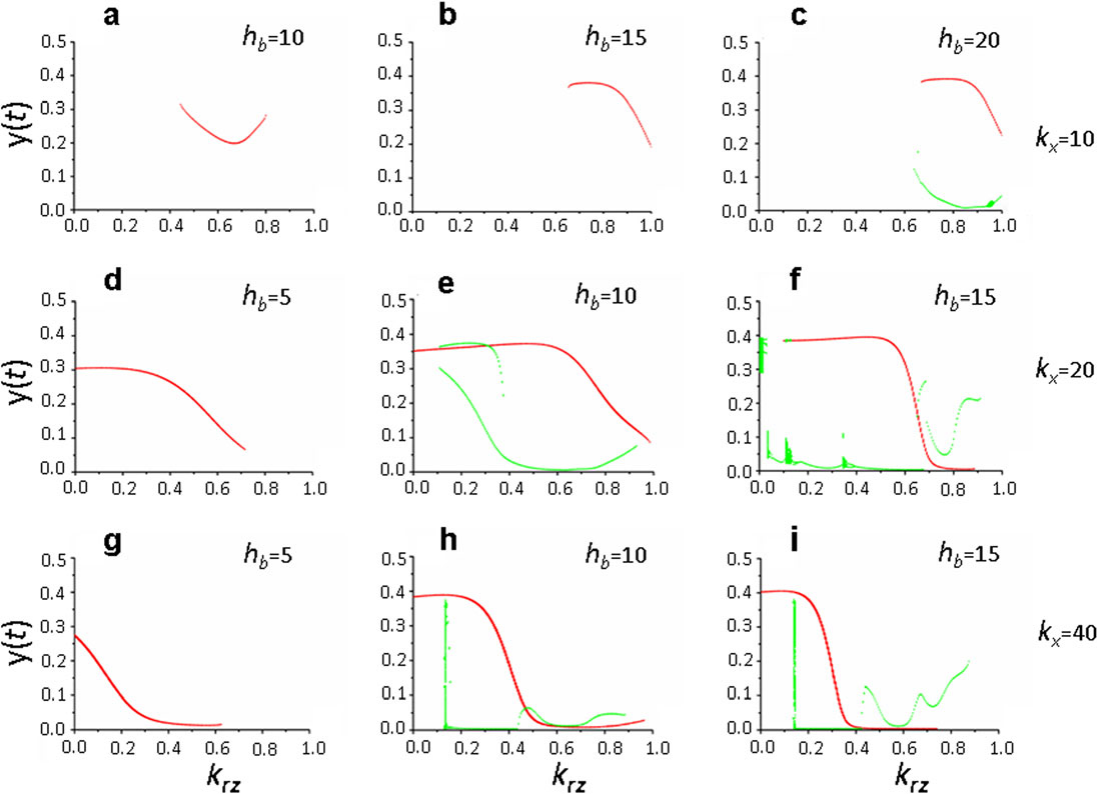
Effect of the complexity of the mTOR signaling pathway (*h*_*b*_) and the rate of synthesis and incorporation of de novo synthesized proteins into the postsynaptic membrane (*k*_*x*_) on the dynamics of the local translation system depending on the recycling parameter *k*_*rz*_. Bifurcation diagram constructed at the intersection of the trajectory (*х*(*t*), *y*(*t*)) with the Poincaré map and *х*(*t*) = 1.5 (**a**), *х*(*t*) = 2.3 (**b**), *х*(*t*) = 2.5 (**c**), *х*(*t*) = 1 (**d**, **g**), *х*(*t*) = 2.0 (**e**, **f**, **h**, **i**), in the phase space (*x,y,z*). Parameter values: *h*_*x*_ = 2, *K*_*a*_ = 1, *k*_*b*_ = 200, *k*_*x*_ = 10 (**a**-**c**), *k*_*x*_ = 20 (**d**-**f**), *k*_*x*_ = 40 (**g**-**i**), *h*_*b*_ = 5 (**d**,**g**), *h*_*b*_ = 10 (**a**, **e**, **h**), *h*_*b*_ = 15 (**b**, **f**, **i**); *τ*_*а*_ = 1, *τ*_*b*_ = 2, *τ*_*r*_ = 3, *τ*_*e*_ = 3. Values of the remaining parameters are shown in the basic set (2)

It has been previously shown that dynamic state of a local translation system at the activated synapse significantly depends on the *h*_*b*_ parameter value, which determines the non-linearity of the signal influence on FMRP phosphorylation processes implemented via the mTOR signaling pathway [9]. The higher the value of *h*_*b*_ parameter, the more complex, quasi-periodic and chaotic regimes of receptor protein synthesis occurred in the system of local translation. Figure 10 shows that in model (1), which considers the recycling contribution to maintaining a pool of active receptor proteins on the postsynaptic membrane, manifestation of this pattern substantially depends on the *k*_*x*_ parameter value, which determines the maximum rate of FMRP-dependent receptor protein synthesis and their incorporation into the membrane.

It was found that at minimum values of both parameters *h*_*b*_ = 5 and *k*_*x*_ = 10, only stationary solutions were observed in model (1), which, with increasing *h*_*b*_ (Table 1, Fig. 10a-c), transform into cyclic ones and this transition occurs at values of recycling parameter *k*_*rz*_ > 0.5. With an increasing *k*_*x*_ values (Table 1, Fig. 10d, g), a transition to cyclic solutions was also observed, but at different values of the recycling parameter *k*_*rz*_ < 0.7. With simultaneous increase in the values of both parameters in model (1), a transition to multiple periodic (Fig. 10e) and even more complex solutions (Fig. 10f, g, h) occurs over almost the entire range of values of the recycling parameter *k*_*rz*_, except for those close to *k*_*rz*_ = 1 (Fig. 10f, i). It should be noted that pattern shown in Fig. 10 is manifested only when the effect of a glutamate-specific signal on the activation of the local translation is low, that is, at *K*_*a*_ = 1. At higher values of this parameter over almost the entire range of values of parameters *h*_*b*_ and *k*_*x*_, except for *k*_*x*_ = 40 (Fig. 9), there is a transition to simpler solutions in system (1) - stationary or periodic (see Additional file 4, Fig. S4), the peculiarities of which are described in sections 3.3 (Fig. 7) and 3.4 (Fig. 8d-f).

## Discussion

Today, there is strong evidence that rapid and well-coordinated changes in the quantitative and qualitative aspects of proteins at the synapse ensure its dynamic plasticity in response to external stimuli and underlie the learning and memory processes [1, 3, 12, 21–24]. Disruption of the local translation control at synapse is associated with various neuropsychiatric diseases, including ASD (autism spectrum disorders) [6, 7, 25, 26], epilepsy [4, 5], Parkinson’s and Alzheimer’s diseases [5, 27, 28], which are characterized by imbalanced synaptic plasticity and lead to changes in behavior, cognitive abilities and memory. It is possible that synaptopathy, which occurs in multiple sclerosis, is associated with dysfunction of the regulatory components of local translation – mTOR signaling and FMRP [29], which regulates the efficiency of dendritic mRNA translation [12]. The structural and functional organization of FMRP activity regulation includes positive and negative regulatory loops that function in different time ranges [19, 20]. At present, it can be considered proven that these aspects of the structural and functional organization of dynamical systems are absolute determinants of the complex dynamics – periodic [30–32], quasi-periodic [32, 33], chaos [34–36] and even hyperchaos [37, 38]. And, the regulatory circuit of the FMRP-dependent translation at the synapse is not an exception. Its chaotic potential was found to be high, and the duration of the signal transduction via mTOR signaling pathway decreased with increasing chaotic potential [9, 10]. Moreover, this could be the cause of autistic disorders and other neuropsychiatric diseases, since one of the characteristics of these conditions is the hyperactivity of the mTOR signaling pathway [7, 28, 39]. In the light of these results [9, 10] and the importance of a stable proteome for the formation of synaptic plasticity and related learning and memory processes [1–3], the assessment of the impact of individual parameters of the FMRP-dependent local translation at activated synapse on its functioning mode is of particular significance.

To address this issue, we developed a more complex model of local translation at activated synapse, in which a certain density of receptor proteins on the postsynaptic membrane was maintained not only due to de novo synthesized protein, but also as a result of protein recycling; and investigated the impact of a number of parameters on the chaotic potential of the system, the values of which have not been experimentally determined. As a result, it was found that increased contribution of receptor recycling to the total pool of active receptors (*k*_*rz*_) added to reducing the complexity of the dynamic regime of the local translation system, but did not determine it. The type of the regime was largely dependent on the complexity of the mechanism of signal transmission via the mTOR signaling pathway (*h*_*b*_) and its transit time (τ_*b*_), which confirmed the results obtained with earlier, simpler model [9]. Another factor influencing the type of the regime was the impact of glumatergic receptors on translation activation (*K*_*a*_) and the rate of synthesis and incorporation of de novo synthesized proteins into the postsynaptic membrane (*k*_*x*_). Moreover, the effect of these factors turned out to be multidirectional - an increase in the *K*_*a*_ and τ_*b*_ values reduced the complexity of the dynamic regime of translation, while an increase in *k*_*x*_ and *h*_*b*_ - increased it. Furthermore, it was found that the manifestation of these regularities had nonlinear relationship with the complexity of the mechanism of exposure of de novo synthesized proteins to the postsynaptic membrane (*h*_*x*_), the correct evaluation of which is currently difficult. It was found that if this process is linear *h*_*x*_ = 1, then system (1) is sufficiently resistant to changes in other parameters and functions either in an oscillatory or in a stationary regime depending on the contribution of recycling (*k*_*rz*_) to maintaining a pool of active receptors on the membrane (see Figs. 6a and 8a-s, S3). However, such mechanism of stabilization of the synaptic receptor protein synthesis is unlikely, since exposure of de novo synthesized proteins to the postsynaptic membrane involves at least two different mechanisms – post-translational modification of de novo synthesized receptor and its subsequent integration into the membrane via exocytosis, i.e. the complexity of this process is most likely *h*_*x*_ > 1.

Patterns described above are manifested if the exposure of de novo synthesized proteins to the postsynaptic membrane is not linear (*h*_*x*_ > 1) and maximum rate of FMRP-dependent synthesis of de novo receptor proteins and their incorporation into the postsynaptic membrane is relatively low (*k*_*x*_ < 40). However, in general, it is quite difficult to determine the parametric areas of the formation of simple dynamic regimes in system (1), since estimation of most of the parameters is rather arbitrary and their action is multidirectional. In this situation, experimental evaluation of at least one of the varying parameters would substantially clarify the situation.

## Conclusions

Thus, the modeling results demonstrate that a certain contribution of receptor recycling to the maintenance of a membrane protein pool decreases the chaotic potential of the local translation system. However, nature of the de novo receptor protein synthesis at the activated synapse is largely determined by the ratio of the parameters of translation activity and exposure of de novo synthesized receptors to the postsynaptic membrane, as well as by the mechanisms of regulation of these processes.

It can also be noted that for a certain ratio between recycling of receptors (*k*_*rz*_ ~ 0.5) and their de novo synthesis, in system (1), practically over the entire parameter space of *K*_*a*_, *k*_*x*_ and *h*_*b*_, stable stationary solutions occur much less frequently than cyclic ones (see Tables 1 and 2). It creates prerequisites for the assumption that cyclical nature of the local FMRP-dependent translation system at the activated synapse may be its “normal” dynamic state.

## Methods

### Model description

The model describes the simplest regulatory circuit of FMRP-dependent local translation of PSD proteins at glutamatergic synapse in response to mGluR receptor stimulation, depending on the ratio between the delay parameters of translational activation and suppression (Fig. 1). It is assumed in the model that a certain PSD protein density on the postsynaptic membrane of the activated synapse is maintained both by de novo protein synthesis and recycling of membrane proteins.

In the model, the regulatory circuit (Fig. 1) is described by three differential equations with delayed arguments.

1 $$ \left\{\begin{array}{l}\frac{dx}{dt}={f}_x\left(y\left(t-{\tau}_e\right)\right)+{k}_{rz}z\left(t-{\tau}_r\right)-\left({k}_{rx}+{k}_{dx}\right)x,\\ {}\frac{dy}{dt}={f}_a\left(x\left(t-{\tau}_a\right)\right)\left({y}_0-y\right)-\left({f}_b\left(x\left(t-{\tau}_b\right)\right)\right)y\\ {}\frac{dz}{dt}={k}_{rx}x-\left({k}_{rz}+{k}_{dz}\right)z,\end{array}\right.,{f}_{\delta }(x)={k}_{\delta, 0}+\frac{k_{\delta }{\left(\frac{x}{K_{\delta }}\right)}^{h_{\delta }}}{1+{\left(\frac{x}{K_{\delta }}\right)}^{h_{\delta }}},\delta =x,a,b, $$

where *x* is a pool of active receptors, *y* is a pool of active FMRP molecules, *z* is a pool of recycling receptor proteins localized in the endosome; *τ*_*a*_ is a duration between signal generation by the receptor under the influence of glutamate molecule and signal realization via FMRP dephosphorylation; *τ*_*b*_ is a duration between signal generation by the receptor under the influence of glutamate molecule and signal realization via FMRP phosphorylation; τ_*e*_ is a total time of protein translation and its incorporation into the receptor complex; *τ*_*r*_ is the time of the receptor protein recycling, indicates the endosomal residence time of the receptor; *k*_*dx*_ is the rate constant for the degradation of receptor proteins by a mechanism that does not include endocytic recycling; *k*_*dz*_ is the rate constant for the degradation of receptor proteins localized in the endosome; *k*_*rx*_ is the rate constant for the inclusion of membrane receptor into the endosome; *k*_*rz*_ is the efficiency constant for the receptor recycling; *f*_*δ*_(*x*) is the control functions that belong to the class of generalized Hill functions [40] and, in the simplest phenomenological form, describe activation (*δ* = *a*) and suppression (*δ = b*) of local translation, as well as incorporation of de novo synthesized receptor proteins into the postsynaptic membrane (*δ = х*); parameters *K*_*δ*_ have the dimensionality of concentration and determine the impact of glutamate-specific signal on activation (*δ* = *a*), suppression (*δ = b*) of local translation, and incorporation of de novo synthesized receptor proteins into the membrane, respectively; parameters *h*_*δ*_ are dimensionless Hill coefficients and determine the nonlinearity of the signal influence on the phosphorylation (*δ = b*) and dephosphorylation of FMRP (*δ* = *a*), as well as incorporation of de novo synthesized receptor proteins into the membrane (*δ = х*); *k*_*x,0*_ and *k*_*x*_ are the constitutive and maximum FMRP-dependent rates for de novo receptor protein synthesis and their incorporation into the postsynaptic membrane, respectively; *k*_*а,*0_ and *k*_*b,*0_ are the rates of background processes (constitutive) of FMRP dephosphorylation and phosphorylation, respectively; *k*_*а*_ and *k*_*b*_ are the– maximum rates of signal-dependent processes of FMRP dephosphorylation and phosphorylation.

In the model, recycling is described by the member *k*_*rz*_*z*(*t* − *τ*_*r*_) of the right side of the first equation: when *k*_*rz*_ = 0, recycling is absent; if *k*_*rz*_ > 0, then part of the proteins *x* from the endosomal pool z is returned to the membrane (recycled). The model is a development of the previously published model [9, 10].

### Estimation of the model parameters

Estimation of the model parameters was carried out by using available experimental data or fundamental biological considerations when specific data were not available.

#### Estimation of delay parameters τ

Delay parameters *τ*_*а*_ and *τ*_*b*_ define time intervals between signal acquisition by the cell membrane receptor and the moment of its effect on translation. Their values were estimated using data from Narayanan et al. [19, 20]. In the calculations, *τ*_*а*_ value was considered equal to one minute [20], and *τ*_*b*_ value varied in the [2, 5] minutes interval [19].

Delay parameter *τ*_*e*_ defines the time interval between initiation of a new protein molecule translation and assembly of a functional cell surface receptor containing this molecule. That is, delay parameter *τ*_*e*_ is the sum of translation elongation time and the time of receptor assembly. The approximate elongation time was estimated from the average translation elongation rate in eukaryotes (~ 6 codons/sec) [41, 42] and the length of mGluR1 receptor protein amino acid sequence (~ 1200 amino acid residues). As a result, average protein synthesis time of 3 min was obtained.

The duration of inclusion of de novo synthesized protein into the membrane was estimated using data from Sharma et al. [43] based on the rate of inclusion of PSD proteins into the membrane, which ranged from 3.3 to 9.7 min. As a result, in the calculations, *τ*_*e*_ value varied in the [3, 20] minutes interval.

The recycling parameter *τ*_*r*_ indicates the time from the moment of receptor insertion into the endosome due to endocytosis to the initiation of receptor return to the membrane as a result of exocytosis, that is, equals to the endosomal residence time. There are no direct data to evaluate this parameter. An indirect assessment of the physiological range of values of this parameter at the activated synapse was made based on data analysis for AMPAR receptors obtained from cell cultures [44–46].

Thus, according to Biou et al. [46], 10% of the receptor molecules that have left the membrane, returned within 10 min. According to data [44, 45], time constant for endocytosis of AMPAR receptor under synaptic activation was ~ 5 min.

Considering these data, in the model, parameter *τ*_*r*_ varied in the [3, 15] minutes interval.

#### Estimation of parameters h_а_, h_b_ and h_х_

Hill coefficients *h*_*а*_, *h*_*b*_ and *h*_*х*_ reflect the nonlinearity of the system and are dimensionless quantities. In the absence of specific data on the mechanisms of activation and suppression of translation, as well as mechanisms of integration of de novo synthesized receptors into the membrane, estimation of their values was rather arbitrary. Nevertheless, proceeding from the fact that phosphatase PP2A dephosphorylates FMRP without intermediators, thus ensuring activation of translation [20, 47], we considered the nonlinearity of this process to have the minimum value *h*_*a*_ = 1.

Suppression of local translation is achieved by S6 kinase-mediated phosphorylation of three amino acid residues of FMRP [19, 47, 48] via the mTOR pathway with a number of intermediate phosphorylation steps (Fig. 1). Some components of this pathway (Akt, TSC2, mTOR) have multiple phosphorylation sites [49]. This suggests that suppression of translation is much more complex and non-linear process as compared with activation of translation. In the model, we considered values of *h*_*b*_ from 5 to 20.

Integration of de novo synthesized receptor proteins into the postsynaptic membrane occurs via exocytosis, however little is known about the location, kinetics, regulation, or molecules involved in postsynaptic exocytosis [50].

Exposure of de novo synthesized receptor proteins to the membrane may be accompanied by posttranslational modifications and multimerization, because of which certain level of nonlinearity may occur. We examined the *h*_*x*_ parameter values from 1 to 3.

#### Estimation of parameters K_a_, K_b_

Parameters *K*_*a*_ and *K*_*b*_ determine the effectiveness of the influence of glutamatergic receptors on activation and suppression of local translation. In essence, they are analogous to the apparent Michaelis constants and are measured in units of concentration. Due to the lack of specific data, we assumed that values of *K*_*a*_ and *K*_*b*_ are proportional to the concentration of receptors on the membrane and, consequently, are comparable in order of magnitude with the number of receptors on the PSD membrane. In addition, proceeding from the fact that activation precedes inhibition, it can be assumed that activation not only takes place prior to inhibitory processes, but also is at least not less efficient. Hence, we consider the inequality *K*_*a*_ ≤ *K*_*b*_. For definiteness, 1 ≤ *K*_*b*_*/K*_*a*_ ≤ 10 was considered in the calculations.

### Calculations

Calculations were carried out on the computer complex of “Data Center of FEB RAS” (Khabarovsk, http://lits.ccfebras.ru), and the Information and Computing Center of Novosibirsk State University (http://www.nusc.ru). The method of integrating the systems of differential equations with delayed arguments was described previously in [37, 38].

Unless specifically indicated, estimation of the effect of recycling on the dynamic properties of the system (1) was carried out with a set of parameter values, which we defined as basic:

2 $$ {\displaystyle \begin{array}{l}{k}_{a0}=0.0,{k}_a=1.0,{K}_a=1.0,{h}_a=1.0,{y}_0=1.0,{k}_{b0}=1.0,{k}_b=200.0,{K}_b=3.0,{h}_b=15.0,{h}_x=3,\\ {}{k}_x=40,{k}_{dx}=0.1,{k}_{rx}=0.9,{k}_{dz}=0.8,{k}_{rz}=0.2,\kern0.5em {\tau}_a=1,\kern0.5em {\tau}_b=1,\kern0.5em {\tau}_r=10,\kern0.5em {\tau}_e=15\end{array}} $$

The Cauchy problem was solved for the initial functions:

$$ x\left(t-\tau \right)=2.5,y\left(t-\tau \right)=0.5,z\left(t-\tau \right)=5. $$

Analysis of the system (1) was carried out with the following variation limits of the parameters:

*h*_*x*_ = 1,2,3, *h*_*b*_ = 5,10,15,20 *k*_*x*_ = 10,20,40, *k*_*b*_ = 100,200, *K*_*a*_ = 1,2,3. As for the variation of the parameters *k*_*dz*_ and *k*_*rz*_, *k*_*rz*_ was changed in the interval [0,1] and *k*_*dz*_ = 1-*k*_*rz*_.

Delayed arguments were varied *τ*_*b*_ = 1–5, *τ*_*e*_ = 3–20, *τ*_*r*_ = 3–15. The rest of the parameters had constant values.

### Methods of estimating the parametric areas of chaos implementation

To estimate the values of the parameters for which chaotic dynamics is predicted for equation (1) with equal values of the delayed arguments, an empirical criterion was applied [51]. There is no corresponding criterion for unequal values of the delayed arguments. Therefore, the chaotic dynamics was studied by numerical analysis, which was determined using two criteria: the sensitivity of solutions with respect to initial data, and the special form of the Poincaré map generated by the solution. Let us give a brief description.

#### Chaos criterion for the oscillatory dynamics

The sensitivity of the oscillatory dynamics was calculated as the difference between two trajectories of one variable, calculated by two identical models that start at zero time point with the initial functions that vary by a “small” value. If, as we iterate over values, the difference became comparable with the fluctuation amplitude, the dynamics was concluded to be sensitive to initial data.

#### Poincaré map

A characteristic form of the Poincaré map (the so-called succession map) allows to explicitly identify the motion pattern in the system. Thus, a cyclic trajectory generates a Poincaré map consisting of a finite number of points through which the trajectory passes with certain regularity. A quasicyclic trajectory is mapped onto a plane as a set of closed curves. The Poincaré map corresponding to a strange attractor represents an unordered infinite set of points. The method for constructing the Poincaré map has been previously described [37, 38].

## Supplementary information

**Additional file 1: Figure S1.** Dynamic regimes of system (1) depending on the FMRP phosphorylation rate (*k*_*b*_) and the influence of the glutamate-specific signal on the activation of translation (*K*_*a*_).

**Additional file 2: Figure S2.** Dynamic regimes of system (1) depending on the rates of signal-dependent FMRP phosphorylation (*k*_*b*_) and FMRP-dependent synthesis of receptor proteins and their incorporation into the membrane (*k*_*x*_).

**Additional file 3: Figure S3.** Dynamic regimes of system (1) given that the mechanism of exposure of de novo synthesized proteins to the postsynaptic membrane is linear *h*_*x*_ = 1.

**Additional file 4: Figure S4.** Dynamic regimes of system (1) depending on the complexity and non-linearity of mTOR (*h*_*b*_) and the influence of the glutamate-specific signal on translation activation (*K*_*a*_).

## Data Availability

All data generated or analyzed during this study are included in this published article and its supplementary information files.

## Abbreviations

FMRP: Fragile X Mental Retardation Protein
mTOR: Mammalian target of rapamycin
mGluR: Metabotropic glutamate receptor
PSD: Postsynaptic density proteins
TSC: Tuberous sclerosis complex
ASD: Autism spectrum disorders
