# Supplementary material for: On the dynamical aspects of local translation at the activated synapse
Source: BMC Bioinformatics. 2020 Sep 14;21(Suppl 11):258. doi: 10.1186/s12859-020-03597-0 (PMC7488754; doi:10.1186/s12859-020-03597-0)
Supplement: Supplementary file 1 — Additional file 1: Figure S1. Dynamic regimes of system (1) depending on the FMRP phosphorylation rate (kb) and the influence of the glutamate-specific signal on the activation of translation (Ka). [file 12859_2020_3597_MOESM1_ESM.pdf]

Bifurcation diagrams of the dynamic regimes of *de novo* protein synthesis at the activated synapse depending on the recycling contribution to the maintenance of the pool of active receptors on the postsynaptic membrane (k_{rz}).

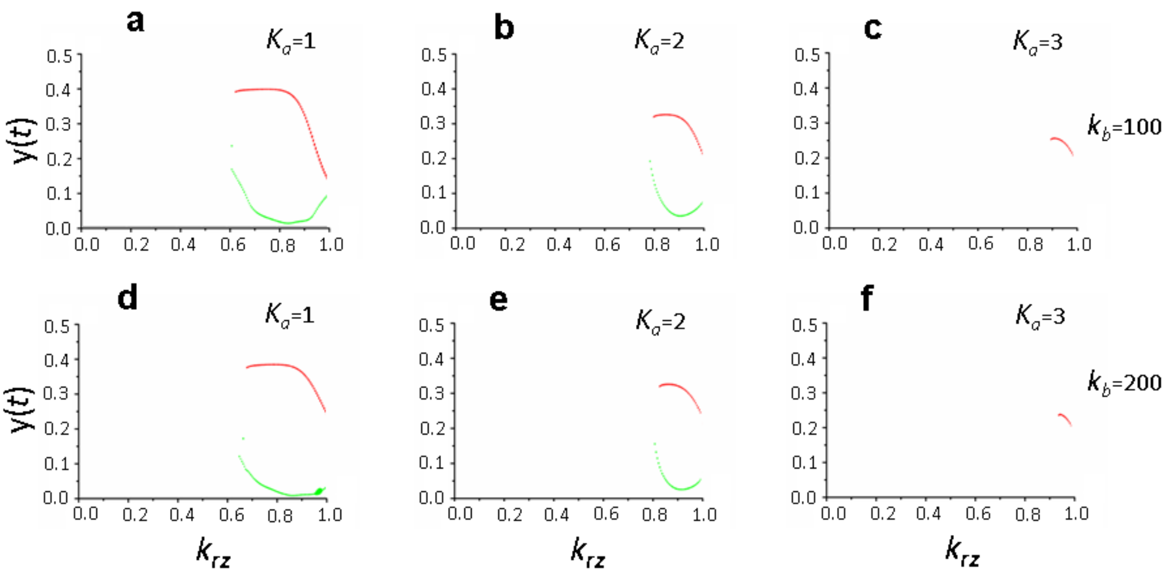

Fig. S1. **Dynamic regimes of system (1) depending on the FMRP phosphorylation rate (k_b) and the influence of the glutamate-specific signal on the activation of translation (K_a).** Bifurcation diagram constructed at the intersection of the trajectory ($x(t)$, $y(t)$) with the Poincaré map $x(t)$=2.50 in the phase space (x,y,z). Parameter values: h_x=2, h_b=20, k_x=10, τ_a=1, τ_b=2, τ_r=3, τ_e=3 (a-f); k_b=100 (a-c), k_b=200 (d-f), K_a=1 (a,d), K_a=2 (b,e), K_a=3 (c,f Values of the remaining parameters are shown in the basic set (2). Different colors indicate different branches of the system (1) solutions.
