# Supplementary material for: On the dynamical aspects of local translation at the activated synapse
Source: BMC Bioinformatics. 2020 Sep 14;21(Suppl 11):258. doi: 10.1186/s12859-020-03597-0 (PMC7488754; doi:10.1186/s12859-020-03597-0)
Supplement: Supplementary file 2 — Additional file 2: Figure S2. Dynamic regimes of system (1) depending on the rates of signal-dependent FMRP phosphorylation (kb) and FMRP-dependent synthesis of receptor proteins and their incorporation into the membrane (kx). [file 12859_2020_3597_MOESM2_ESM.pdf]

Bifurcation diagrams of the dynamic regimes of *de novo* protein synthesis at the activated synapse depending on the recycling contribution to the maintenance of the pool of active receptors on the postsynaptic membrane (k_{rz}).

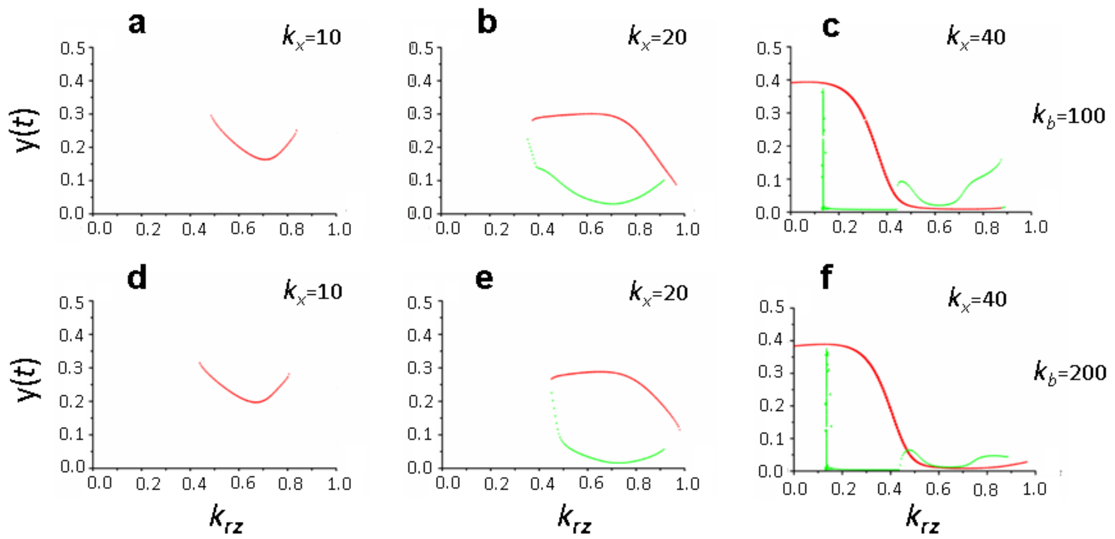

Fig. S2. Dynamic regimes of system (1) depending on the rates of signal-dependent FMRP phosphorylation (k_b) and FMRP-dependent synthesis of receptor proteins and their incorporation into the membrane (k_x). Bifurcation diagram constructed at the intersection of the trajectory ($x(t)$, $y(t)$) with the Poincaré map $x(t)$=1.70 (a,d) and $x(t)$=2 (b,c,e,f) in the phase space (x,y,z). Parameter values: K_a=1, h_x=2, h_b=10, τ_a=1, τ_b=2, τ_r=3, τ_e=3 (a-f); k_x=10 (a,d), k_x=20 (b,e), k_x=40 (c,f), k_b=100 (a-c), k_b=200 (d-f). Values of the remaining parameters are shown in the basic set (2).
