# Supplementary material for: On the dynamical aspects of local translation at the activated synapse
Source: BMC Bioinformatics. 2020 Sep 14;21(Suppl 11):258. doi: 10.1186/s12859-020-03597-0 (PMC7488754; doi:10.1186/s12859-020-03597-0)
Supplement: Supplementary file 3 — Additional file 3: Figure S3. Dynamic regimes of system (1) given that the mechanism of exposure of de novo synthesized proteins to the postsynaptic membrane is linear hx = 1. [file 12859_2020_3597_MOESM3_ESM.pdf]

Bifurcation diagrams of the dynamic regimes of *de novo* protein synthesis at the activated synapse depending on the recycling contribution to the maintenance of the pool of active receptors on the postsynaptic membrane (k_{rz}).

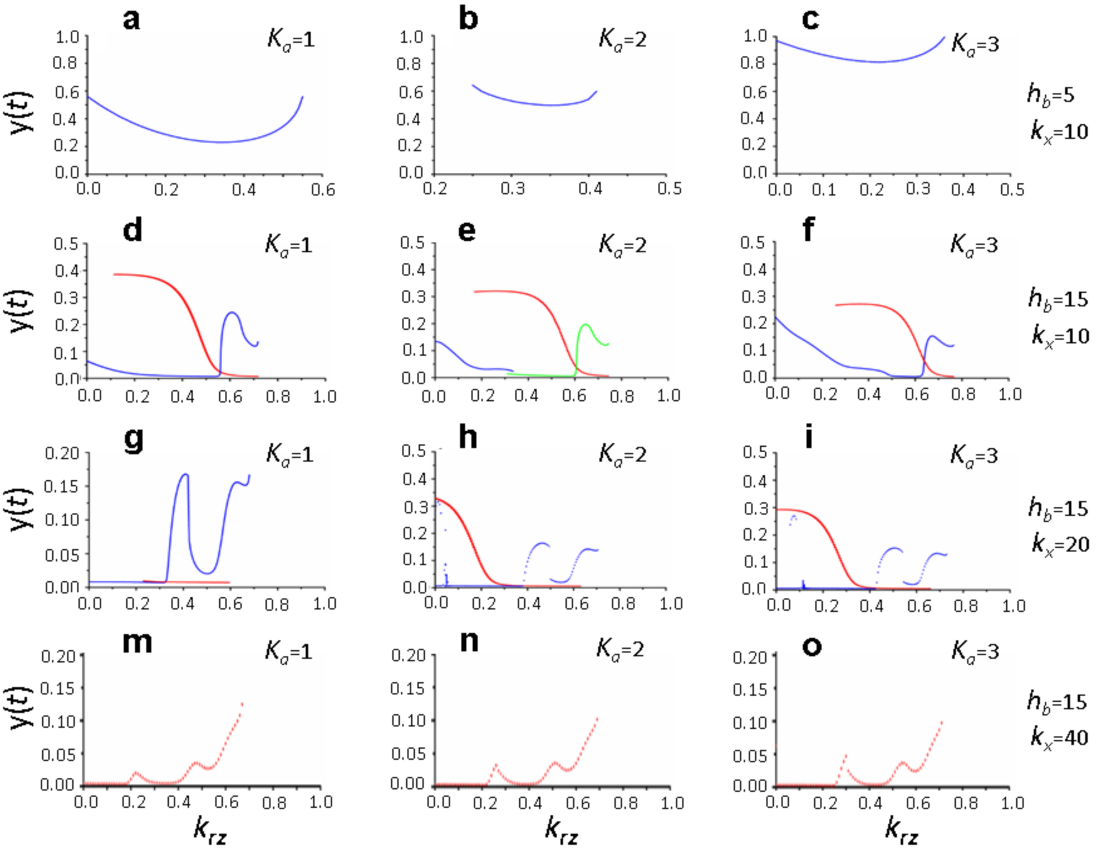

Fig. S3. Dynamic regimes of system (1) given that the mechanism of exposure of *de novo* synthesized proteins to the postsynaptic membrane is linear h_x=1. Bifurcation diagram constructed at the intersection of the trajectory ($x(t)$, $y(t)$) with the Poincaré map $x(t)$=2 in the phase space (x,y,z). Parameter values: τ_a=1, τ_b=2, τ_r=3, τ_e=3, h_x = 1 (a-o); h_b = 5, k_x=10, k_b=100 (a-c); h_b = 15, k_x=10, k_b=100 (d-f); h_b = 15, k_x=20, k_b=100 (g-i); h_b = 15, k_x=40, k_b=200 (m-o); K_a=1 (a, d, g, m), K_a=2 (b, e, h, n), K_a=3 (c, f, i, o). Values of the remaining parameters are shown in the basic set (2). Different colors indicate different branches of the system (1) solutions.
