# Supplementary material for: On the dynamical aspects of local translation at the activated synapse
Source: BMC Bioinformatics. 2020 Sep 14;21(Suppl 11):258. doi: 10.1186/s12859-020-03597-0 (PMC7488754; doi:10.1186/s12859-020-03597-0)
Supplement: Supplementary file 4 — Additional file 4: Figure S4. Dynamic regimes of system (1) depending on the complexity and non-linearity of mTOR (hb) and the influence of the glutamate-specific signal on translation activation (Ka). [file 12859_2020_3597_MOESM4_ESM.pdf]

**Bifurcation diagrams of the dynamic regimes of *de novo* protein synthesis at the activated synapse depending on the recycling contribution to the maintenance of the pool of active receptors on the postsynaptic membrane ($k_{rz}$).**

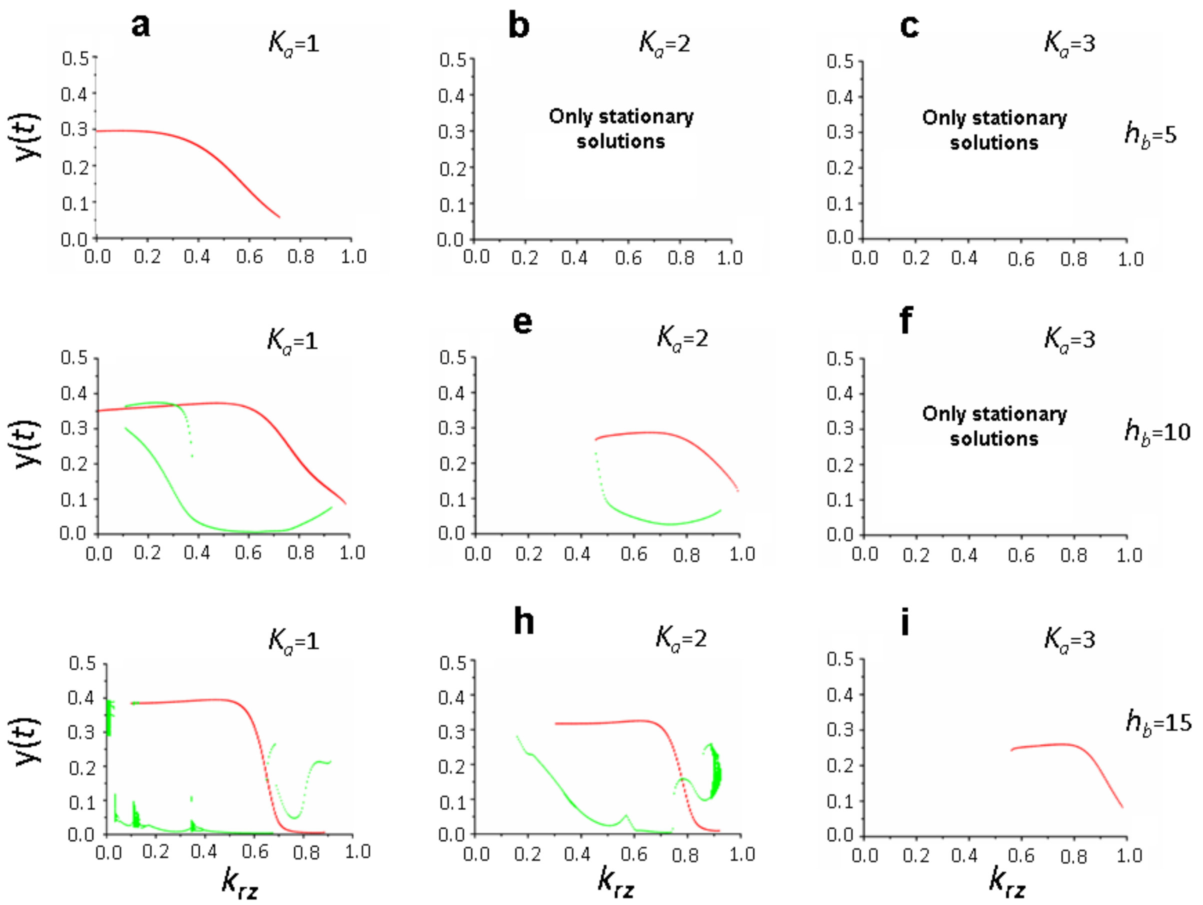


**Fig. S4. Dynamic regimes of system (1) depending on the complexity and non-linearity of mTOR ($h_b$) and the influence of the glutamate-specific signal on translation activation ($K_a$).**

Bifurcation diagram constructed at the intersection of the trajectory ($x(t)$, $y(t)$) with the Poincaré map $x(t)$=1(a-c), $x(t)$=2 (d-i) in the phase space ($x,y,z$). Parameter values: $h_x$ = 2, $k_x$=20, $k_b$=200, $\tau_a$=1, $\tau_b$=2, $\tau_r$=3, $\tau_e$=3 (a-i); $h_b$ = 5 (a-c), $h_b$ = 10 (d-f), $h_b$ = 15 (g-i), $K_a$=1 (a, d, g), $K_a$=2 (b, e, h), $K_a$=3 (c, f, i). Values of the remaining parameters are shown in the basic set (2). Different colors indicate different branches of the system (1) solutions.
